# Open access individual finger movement dataset with fNIRS

**DOI:** 10.3389/fnhum.2026.1747655

**Published:** 2026-03-13

**Authors:** Haroon Khan, Hammad Nazeer, Peyman Mirtaheri

**Affiliations:** 1 Department of Mechanical, Electronics and Chemical Engineering, OsloMet—Oslo Metropolitan University, Oslo, Norway; 2 Department of Mechatronics and Biomedical Engineering, Air University, Islamabad, Pakistan

**Keywords:** brain-computer interface (BCI), dataset, functional near-infrared spectroscopy (fNIRS), individual finger movements, open access

## 1 Introduction

Functional near-infrared spectroscopy (fNIRS) is a neuroimaging modality with an acceptable spatial and temporal resolution that enables continuous, non-invasive, portable, safe, and affordable monitoring of blood oxygenation and blood volume (Pinto-Orellana et al., 2024). The theory behind the fNIRS measurements is neurovascular coupling and optical spectroscopy. An increased neuronal activation demands higher oxygen consumption to fulfill neuronal tissue demands (Pinto-Orellana et al., 2024; Boas et al., 2003). Most of the biological tissues are transparent to light in the near-infrared range (700–900 nm). Hence, relatively little scattering occurs when NIRS light is transmitted into the tissue. The relative change in absorption and back-scatter photons from oxygenated hemoglobin (HbO) and deoxygenated hemoglobin (HbR) chromophores provides information about neural activity through a process known as neurovascular coupling. In summary, fNIRS monitors brain hemodynamics in a safe, easy, low-cost, portable, and low-noise manner (compared to functional magnetic resonance imaging), making it an attractive tool for neuroimaging and its applications (Ferrari and Quaresima, 2012).

The use of fNIRS in the field of brain-computer interface (BCI) is relatively recent, yet is rapidly gaining popularity (Naseer and Hong, 2015; Finnis et al., 2025). In a BCI system, brain activity is decoded and translated into control commands to operate external devices or computers. A typical BCI framework consists of several key stages, including signal acquisition, preprocessing, feature extraction, classification, and control signal generation (Nicolas-Alonso and Gomez-Gil, 2012). Among the various brain regions investigated in fNIRS-based BCIs, the motor cortex and prefrontal cortex are the most extensively studied. The motor cortex is primarily responsible for executing voluntary movements of different body parts. In fNIRS-based paradigms, motor execution tasks commonly involve finger, hand, or foot tapping (Kashou et al., 2016; Bak et al., 2019; Khan et al., 2021, 2024b; Ding et al., 2025). Thumb and little finger movements were classified with an accuracy of 87.5% using Δ*HbO* data (Zafar and Hong, 2020). More recently, deep learning methods have gained traction for handling the classification of these complex finger movements. Using convolutional neural networks (CNNs), left-finger, right-finger, and foot-tapping tasks were classified with a high accuracy of 96.67% (Wickramaratne and Mahmud, 2021). Another recent study distinguished left and right index finger-tapping performed at different frequencies by applying multi-labeling and deep learning techniques (Sommer et al., 2021). Separate labels were assigned to each tapping condition—rest, 80 bpm, and 120 bpm—for both hands. Despite the complexity of this labeling scheme, the deep learning model achieved an average classification accuracy of 81%. Direct comparison across these studies is difficult because they employed different models and finger-tapping paradigms. Nonetheless, the literature consistently highlights that differentiating fine finger-movement patterns using fNIRS remains highly challenging. However, movements involving fine anatomical structures—such as individual finger tapping, particularly within one hand—have received limited attention in fNIRS-based BCIs, with only a few recent studies exploring this aspect (Khan et al., 2021, 2024b,a). Some attempts have been made to classify such fine motor movements using other neuroimaging modalities, including EEG and fMRI (Shen et al., 2014; Liao et al., 2014; Ding et al., 2025). One possible reason for the limited exploration in fNIRS could be its inherent limitations, such as relatively low temporal resolution (1–10 Hz for most commercially available portable systems), depth sensitivity of approximately 1.5 cm (depending on the source–detector distance, typically around 3 cm), and spatial resolution of about 1 cm (Ferrari and Quaresima, 2012).

The dataset was collected with the motivation to explore the potential of fNIRS, in combination with modern machine learning algorithms, for decoding and classifying fine anatomical movements such as individual finger motions. In fNIRS, data acquisition is time-consuming, equipment-intensive, and often limited by laboratory capacity. As a result, publicly available fNIRS datasets typically include a modest number of participants (Luke and McAlpine, 2021; Bak et al., 2019; Khan et al., 2025; von Lühmann et al., 2020; Chen et al., 2023; Ning et al., 2024). However, such advancements could open up a broad range of applications in the field of fNIRS-based BCI. For instance, Ding et al. (2025) developed a real-time robotic hand control system using an EEG-based BCI with individual finger movements. Given the high compatibility between EEG and fNIRS for real-time hybrid BCI systems, integrating both modalities could lead to more reliable and robust BCI applications. Nevertheless, it is hypothesized that motor cortex signals contain valuable information that can be leveraged to enhance control commands through advanced machine learning algorithms. The finger-tapping task is a well-understood and relatively simple motor task with distinct cortical activation patterns, making it a standard paradigm in BCI research (Middendorf et al., 2000). However, even for such tasks, detecting and classifying anatomical structures—such as distinguishing between individual finger movements—remains a challenging and ongoing area of investigation.

Nevertheless, with recent advancements in machine learning algorithms, it has become increasingly feasible to extract intrinsic and independent information from the hemodynamic responses captured by fNIRS. Therefore, this report presents an open-access dataset derived from an individual finger-tapping experiment, designed to facilitate the application and development of advanced algorithms for decoding dexterous movements from fNIRS signals. The dataset will be available for researchers and scholars to perform further analyses, explore new perspectives, and test novel hypotheses related to spatial information processing. The preliminary results demonstrate distinct activation patterns associated with individual finger movements (Khan et al., 2024b). These patterns can be effectively classified using both conventional machine learning approaches (Khan et al., 2021) and deep learning methods (Khan et al., 2024a), achieving accuracies that suggest promising potential for BCI applications. This report further outlines the essential methodological steps employed during the data acquisition of the individual finger-tapping experiment. Additionally, it provides a detailed description of the materials and procedures adopted during data collection, offering valuable insights for researchers aiming to conduct high-quality fNIRS experiments.

## 2 Methodology

### 2.1 Instrumentation

Details of the software and hardware used for data collection are provided in Table 1. The only exception is the dataset labeled S25, which was collected using NIRSport 2 at a sampling rate of 10.1725 Hz. Additionally, the duration of the rest and task blocks for this dataset slightly differs, as will be discussed later in Section 4.

**Table 1:** List of hardware and software used for data collection.

**Sr. no**.	**Hardware/ software name**	**Description**
1	NIRScout	Dual-wavelength (λ_1_ = 760nm, λ_2_ = 850nm) near-infrared diffuse tomographic system (NIRx Medizintechnik GmbH, Germany).
2	NIRStim 4.0	Software for stimulus presentation (NIRx Medizintechnik GmbH, Germany).
3	NIRStar 15.2	Software for data acquisition (NIRx Medizintechnik GmbH, Germany).
4	Sampling rate	3.9063 Hz.
5	Sources/detectors	16 each.

### 2.2 Environment condition

The experiment was conducted in a quiet room to minimize distractions. Laboratory lights were dimmed during data acquisition to reduce the influence of external light on fNIRS measurements. The monitor brightness was set to 50% to further minimize its effect on the recordings. Additionally, an NIRx cap cover was used to shield the optodes from ambient light, ensuring more reliable measurements.

## 3 Participants and demographics

A total of 25 right-handed participants (19 males and six females) took part in the study. The mean age was 30.44 ± 2.6 years for males (range: 25–39 years) and 29.16 ± 2.5 years for females (range: 25–34 years). Handedness was determined based on the participants' self-reported preference for writing with the right hand, consistent with the general definition of handedness as the tendency to preferentially use one hand for uni-manual tasks (Corey et al., 2001). Only right-handed individuals were included to minimize hemispheric variability, as approximately 90% of the population is right-handed with corresponding left-hemisphere dominance. The demographics are further elaborated in the Table 2.

**Table 2:** Demographic information of participants included in the feature extraction study.

**Subject ID**	**Age (years)**	**Hand dominance**	**Education**	**Gender**	**Runs**
S01	32	Right	Graduate	M	2
S02	32	Right	Graduate	M	6
S03	34	Right	Graduate	F	2
S04	30	Right	Graduate	M	5
S05	39	Right	Bachelor	M	3
S06	32	Right	Bachelor	M	3
S07	31	Right	Bachelor	M	2
S08	29	Right	Bachelor	M	3
S09	28	Right	Bachelor	M	3
S10	29	Right	Graduate	M	3
S11	29	Right	Graduate	F	3
S12	30	Right	Graduate	F	2
S13	31	Right	Bachelor	M	3
S14	29	Right	Graduate	M	3
S15	30	Right	Bachelor	M	1
S16	30	Right	Bachelor	M	3
S17	30	Right	Graduate	F	3
S18	29	Right	Graduate	M	2
S19	32	Right	Graduate	M	2
S20	26	Right	Bachelor	M	3
S21	27	Right	Graduate	F	3
S22	25	Right	Graduate	F	3
S23	30	Right	Graduate	M	1
S24	34	Right	Bachelor	M	3
S25	25	Right	Bachelor	M	2
**Total runs**					**69**

Bold value indicates the total number of experimental runs in the entire dataset for all the subjects.

## 4 Experimental design

### 4.1 Experimental paradigm

**Figure 1:**
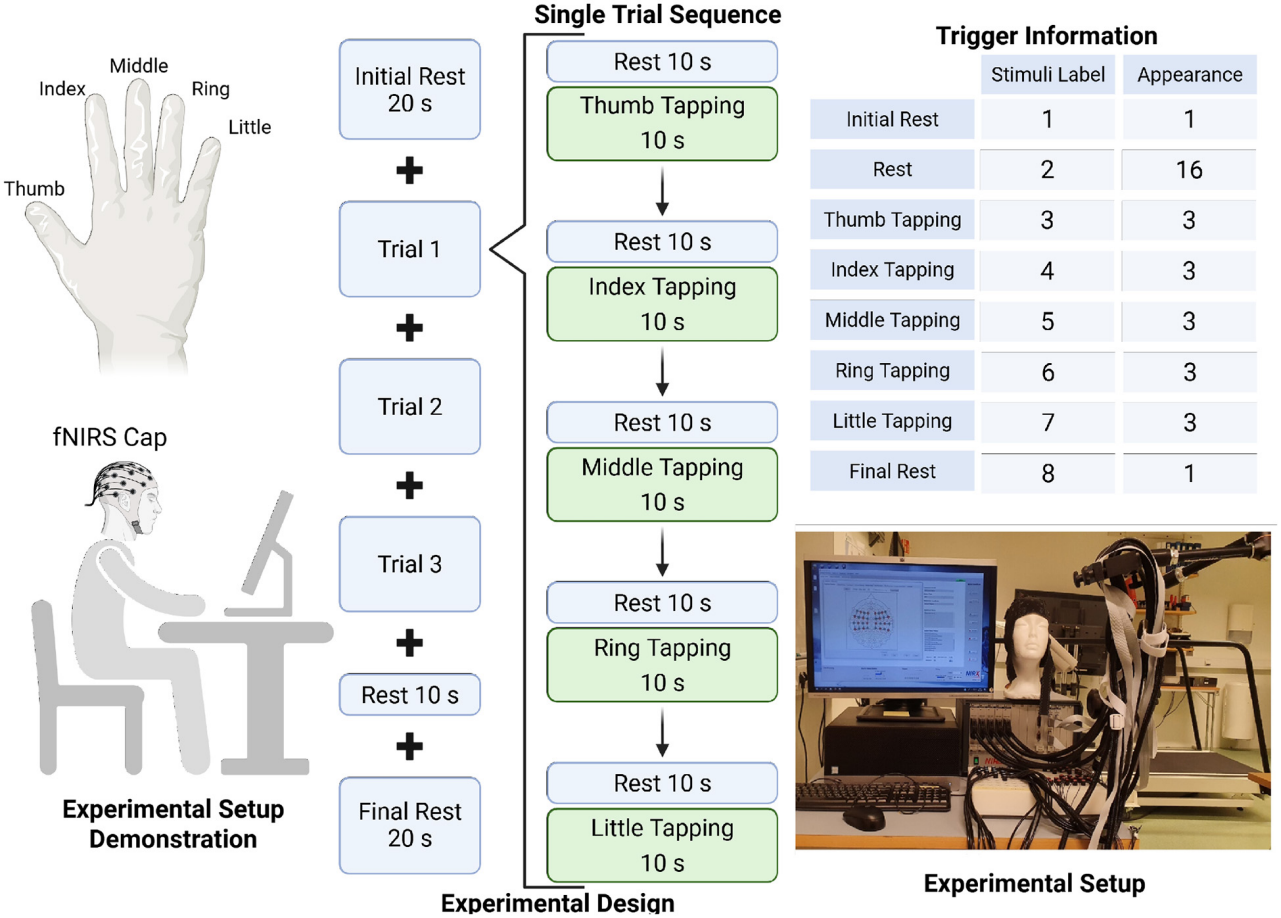
Details of the experimental setup and design, and stimuli information.

The experimental paradigm followed a block design consisting of rest and task blocks (individual finger-tapping), as illustrated in Figure 1. A baseline rest of 20 s was provided before and after the first and last tasks, labeled as Initial Rest and Final Rest, respectively. The intermediate rest blocks were set to 10 s, except for dataset S25, where they were 15 s. Detailed timing information is provided in a .TEXT file accompanying the dataset. Each finger-tapping task lasted 10 s, as shown in the “Single Trial Sequence." A single experiment trial consisted of three repetitions, with each trial containing alternating rest and task blocks. Within a single trial, five blocks of rest and task were presented, with finger-tapping performed sequentially from thumb to little finger. The duration of a single trial was 100 s, and a complete experiment, consisting of three trials, lasted 350 s. Instructions for finger-tapping were displayed on a computer monitor. Trigger labels and their occurrences are also indicated in Figure 1. The finger tapping was performed self-paced.

### 4.2 Participant training and interaction

Before the experiment, participants received detailed instructions regarding the experimental protocol, the duration of the experiment, the number of trials, and other factors that could influence the results. They were instructed to remain calm and avoid any unnecessary movements, including head or body movements, that might affect the measurements. If a participant experienced any discomfort, the experiment was immediately aborted. The total number of experimental repetitions per participant was determined based on their comfort level.

### 4.3 Brain region

**Figure 2:**
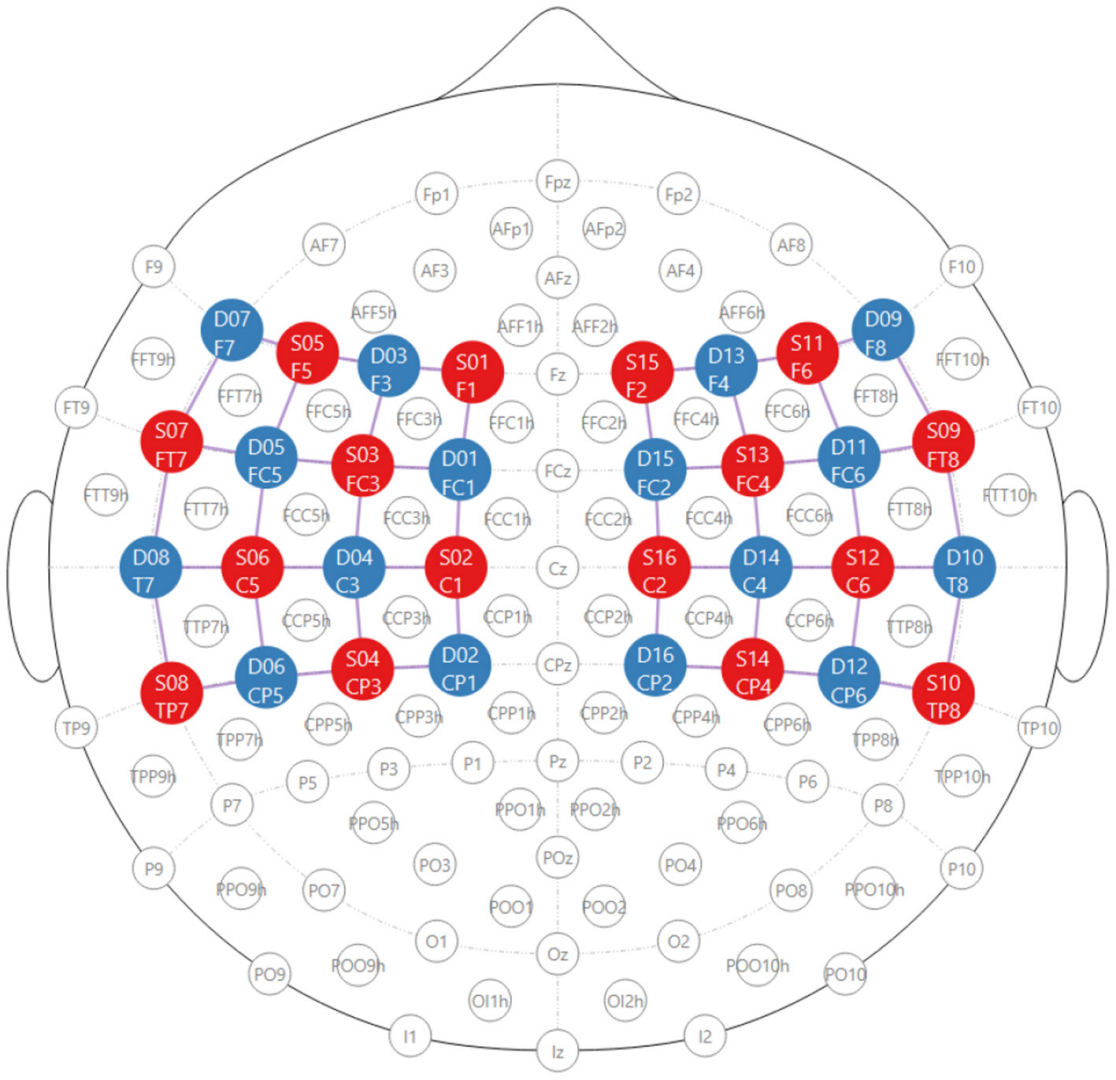
Topographic NIRS montage based on the international 10–10 positioning system. Red circles represent sources (S01–S16), where “S" denotes source and the number indicates the source index. Blue circles represent detectors (D01–D16), where “D" denotes detector and the number indicates the detector index. Purple lines illustrate the NIRS channels of interest formed between source–detector pairs. The layout consists of 16 sources and 16 detectors (32 optodes in total), generating 48 measurement channels with an inter-optode spacing of 3 cm, optimized for motor cortex measurements.

Before the experiment, each participant's head circumference was measured to ensure proper selection of the NIRx cap. The Cz location was identified by marking the midpoint between the nasion and the inion, and the preauricular points on both the left and right sides. Optodes were then placed over the motor cortex following the international 10–10 electrode placement system, as illustrated in Figure 2. The details of channel configuration for both the left (CH01–CH24) and right (CH25–CH48) hemispheres, including the corresponding source–detector pairs, are presented in Table 3.

**Table 3:** fNIRS channel configuration based on the 10–10 international electrode placement system.

**Left hemisphere**			**Right hemisphere**		
**Channel**	**Source**	**Detector**	**Channel**	**Source**	**Detector**
CH01	S01 (F1)	D01 (FC1)	CH25	S09 (FT8)	D09 (F8)
CH02	S01 (F1)	D03 (F3)	CH26	S09 (FT8)	D10 (T8)
CH03	S02 (C1)	D01 (FC1)	CH27	S09 (FT8)	D11 (FC6)
CH04	S02 (C1)	D02 (CP1)	CH28	S10 (TP8)	D10 (T8)
CH05	S02 (C1)	D04 (C3)	CH29	S10 (TP8)	D12 (CP6)
CH06	S03 (FC3)	D01 (FC1)	CH30	S11 (F6)	D09 (F8)
CH07	S03 (FC3)	D03 (F3)	CH31	S11 (F6)	D11 (FC6)
CH08	S03 (FC3)	D04 (C3)	CH32	S11 (F6)	D13 (F4)
CH09	S03 (FC3)	D05 (FC5)	CH33	S12 (C6)	D10 (T8)
CH10	S04 (CP3)	D02 (CP1)	CH34	S12 (C6)	D11 (FC6)
CH11	S04 (CP3)	D04 (C3)	CH35	S12 (C6)	D12 (CP6)
CH12	S04 (CP3)	D06 (CP5)	CH36	S12 (C6)	D14 (C4)
CH13	S05 (F5)	D03 (F3)	CH37	S13 (FC4)	D11 (FC6)
CH14	S05 (F5)	D05 (FC5)	CH38	S13 (FC4)	D13 (F4)
CH15	S05 (F5)	D07 (F7)	CH39	S13 (FC4)	D14 (C4)
CH16	S06 (C5)	D04 (C3)	CH40	S13 (FC4)	D15 (FC2)
CH17	S06 (C5)	D05 (FC5)	CH41	S14 (CP4)	D12 (CP6)
CH18	S06 (C5)	D06 (CP5)	CH42	S14 (CP4)	D14 (C4)
CH19	S06 (C5)	D08 (T7)	CH43	S14 (CP4)	D16 (CP2)
CH20	S07 (FT7)	D05 (FC5)	CH44	S15 (F2)	D13 (F4)
CH21	S07 (FT7)	D07 (F7)	CH45	S15 (F2)	D15 (FC2)
CH22	S07 (FT7)	D08 (T7)	CH46	S16 (C2)	D14 (C4)
CH23	S08 (TP7)	D06 (CP5)	CH47	S16 (C2)	D15 (FC2)
CH24	S08 (TP7)	D08 (T7)	CH48	S16 (C2)	D16 (CP2)

Channels CH01–CH24 correspond to the left hemisphere, and CH25–CH48 correspond to the right hemisphere.

### 4.4 Data processing

**Figure 3:**
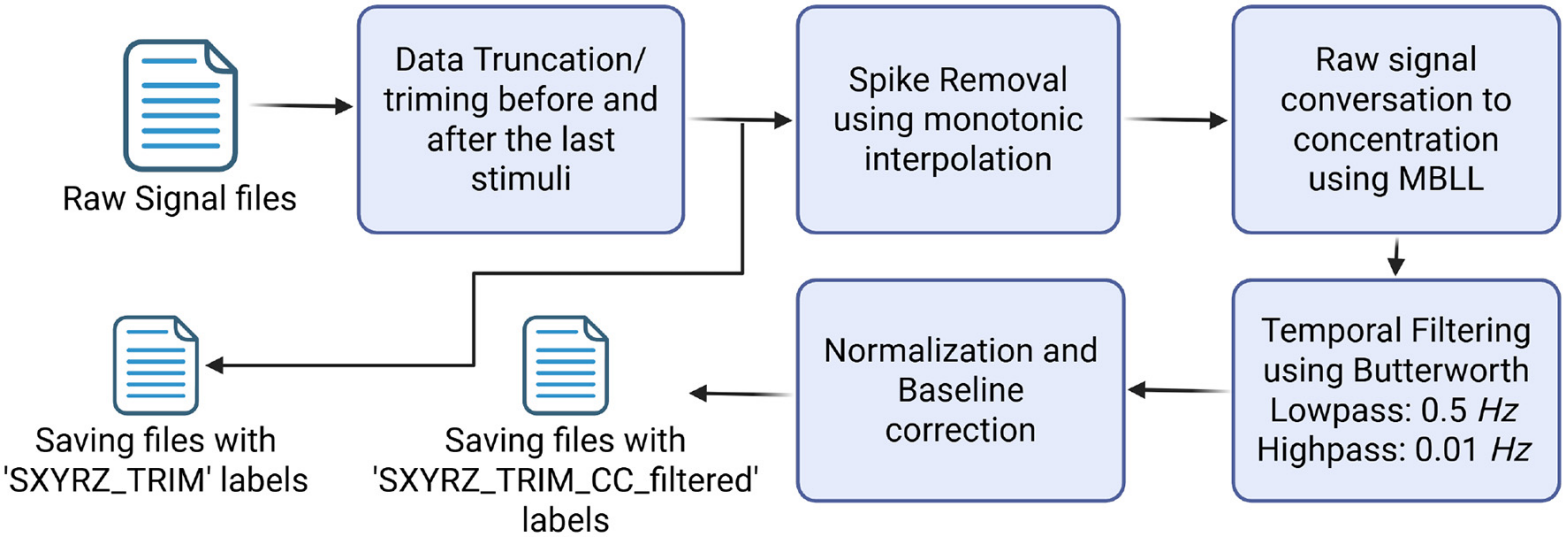
The flow diagram of data processing. The dataset contains files with both processed and unprocessed data.

A basic signal processing pipeline was applied to filter the data. Data processing was performed using the commercial software Satori v2.2 (NIRx Medizintechnik GmbH, Germany). The processing steps included spike removal, conversion of raw intensities into concentration changes, temporal filtering, normalization, and baseline zero adjustment. A Butterworth filter with high-pass 0.01 *Hz* and low-pass 0.5 *Hz* was applied to filter the signals. The overall processing pipeline and the labeling of processed and unprocessed data are illustrated in Figure 3.

### 4.5 Event averages–data visualization

**Figure 4:**
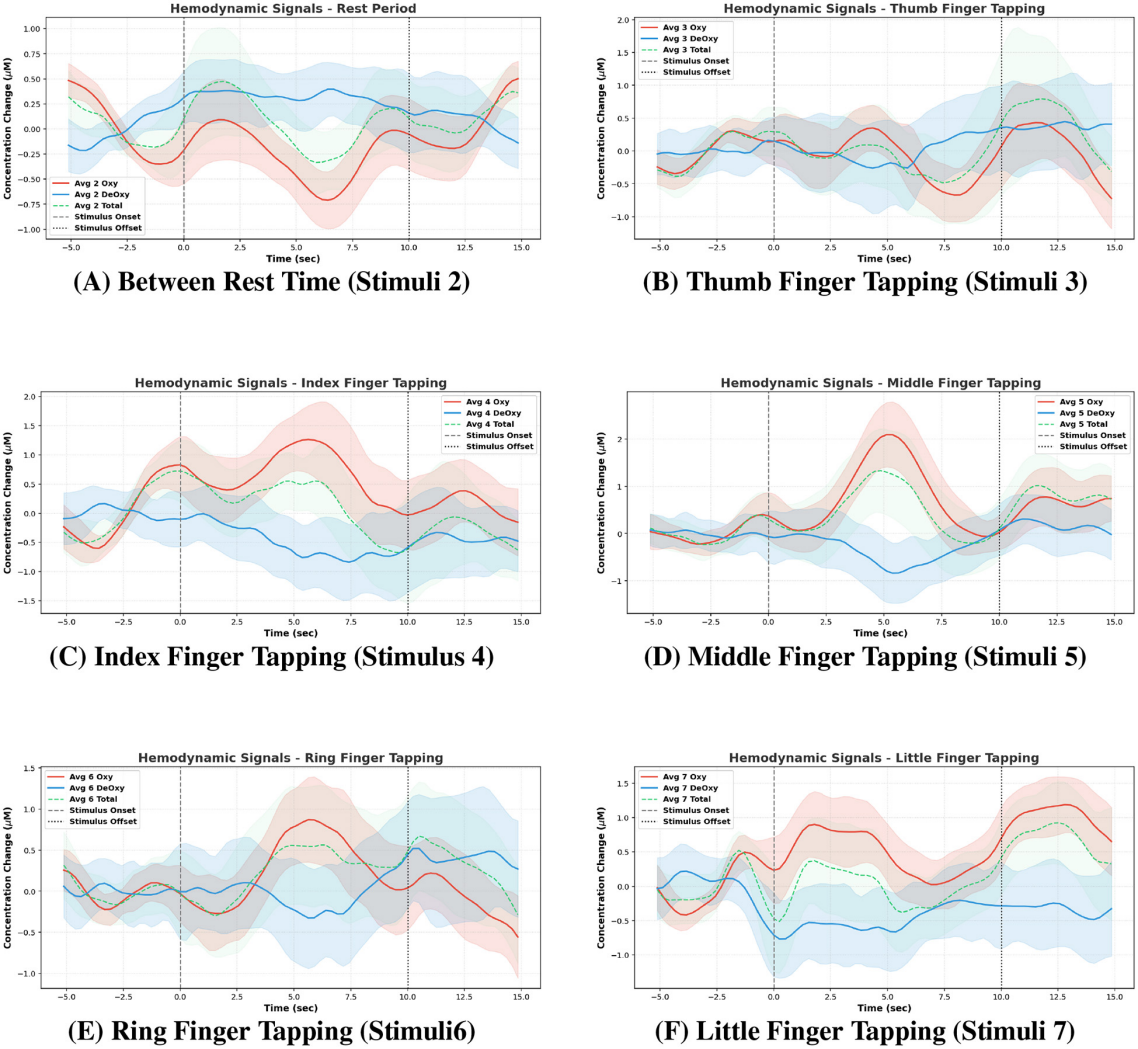
Event-averaged responses for different finger movements for subject (S02). **(B–F)** correspond to individual finger responses—**(B)** thumb finger tapping (stimuli 3), **(C)** index finger tapping (stimulus 4), **(D)** middle finger tapping (stimuli 5), **(E)** ring finger tapping (stimuli 6), and **(F)** little finger tapping (stimuli 7). **(A)** represents the in-between en rest period average response—between rest time (stimuli 2).

The event-averaged responses of all channels, along with their standard deviations for S02 as an example, are shown in the Figure 4. The results clearly demonstrate that different finger movements produce distinct hemodynamic response patterns. The average response during the *rest period* was also plotted to illustrate how the resting-state activity compares with the movement-related changes for each finger.

To compute the average responses, a 5-s pre-stimulus window and a 5-s post-stimulus window were included. This approach captures the behavior of the signals both before and after tapping, providing a clearer understanding of the response dynamics associated with each movement.

## 5 Data structure and format

The dataset was originally collected in the previous fNIRS file formats (.wl1, .w12, .hdr, .avg, _config, _probInfor) and subsequently converted (using Satori v2.2) into the standardized *Shared Near-Infrared Spectroscopy Format* (SNIRF) (Tucker et al., 2023). The dataset comprises recordings from 25 subjects, labeled as SXY, where S denotes the subject identifier and XY ranges from 01 to 25. Here, RZ represents the run index, with R indicating the run number (i.e., repetitions of the experiment for the same subject) and Z ranging from 1 to 6. For example, S02R6_TRIM corresponds to data from Subject 02 during the sixth repetition of the experimental task. Each subject folder contains .SNIRF files named according to the convention SXYRZ_TRIM, which are the unfiltered data in .SNIRF format. The SXYRZ_TRIM_CC_filtered is filtered (according to the pipeline mentioned in Section 4.4) and includes hemoglobin concentration data. The number of repetitions varies across subjects, depending on their individual comfort levels. As mentioned earlier, the data were sampled at 3.90625 Hz, with a total recording duration of 350 s per experimental run, resulting in 1,367 measurement time points. The total number of measurement channels was 48. The dataset includes eight stimulus triggers, as described in the Figure 1. The measurement wavelengths were 760 *nm* and 850 *nm*, enabling the recording of changes in both oxy-hemoglobin and deoxy-hemoglobin, as indicated in the Table 1. Minor deviations from the standard experimental design were made for two subjects, as documented in the experimental notes file included with the dataset. **Note:** These files contain the raw, unfiltered data. Other relevant information regarding the hardware configuration and calibration procedures is available from the authors upon request.

## 6 Conclusion

In conclusion, the paper presents a functional near-infrared spectroscopy (fNIRS) dataset from 25 healthy subjects performing individual right-hand finger-tapping tasks (thumb, index, middle, ring, and little fingers). Preliminary analysis demonstrates that motor cortex fNIRS signals encode intrinsic and independent information about these fine movements and can be classified with significant accuracy using modern machine learning methods. This dataset enables the development and validation of algorithms for fine motor movement classification and supports fNIRS-based brain-computer interface (BCI) research. Moreover, because finger-tapping tasks are common across multiple brain imaging modalities, the dataset provides opportunities for cross-modality comparisons of neural responses to fine motor actions.

## Data Availability

The datasets presented in this study can be found in online repositories. The names of the repository/repositories and accession number(s) can be found at: https://figshare.com/s/6298861b1dc6be936d73?file=59576999.
